# Expression Patterns of Killer Cell Immunoglobulin-Like Receptors (KIR) of NK-Cell and T-Cell Subsets in Old World Monkeys

**DOI:** 10.1371/journal.pone.0064936

**Published:** 2013-05-22

**Authors:** Meike Hermes, Christina Albrecht, Annette Schrod, Markus Brameier, Lutz Walter

**Affiliations:** 1 Primate Genetics Laboratory, German Primate Center, Leibniz Institute for Primate Research, Göttingen, Germany; 2 Pathology Unit, German Primate Center, Leibniz Institute for Primate Research, Göttingen, Germany; New York University, United States of America

## Abstract

The expression of killer cell immunoglobulin-like receptors (KIR) on lymphocytes of rhesus macaques and other Old World monkeys was unknown so far. We used our recently established monoclonal anti-rhesus macaque KIR antibodies in multicolour flow cytometry for phenotypic characterization of KIR protein expression on natural killer (NK) cells and T cell subsets of rhesus macaques, cynomolgus macaques, hamadryas baboons, and African green monkeys. Similar to human KIR, we found clonal expression patterns of KIR on NK and T cell subsets in rhesus macaques and differences between individuals using pan-KIR3D antibody 1C7 and antibodies specific for single KIR. Similar results were obtained with lymphocytes from the other studied species. Notably, African green monkeys show only a low frequency of KIR3D expressed on CD8^+^ αβT cells. Contrasting human NK cells are KIR-positive CD56bright NK cells and frequencies of KIR-expressing NK cells that are independent of the presence of their cognate MHC class I ligands in rhesus macaques. Interestingly, the frequency of KIR-expressing cells and the expression strength of KIR3D are correlated in γδ T cells of rhesus macaques and CD8^+^ αβT cells of baboons.

## Introduction

Killer cell immunoglobulin-like receptors (KIR) form a family of diverse type I receptors with variable numbers of extracellular immunoglobulin (Ig)-like domains. Depending on the type of transmembrane and cytoplasmic regions, KIRs are classified as either inhibitory or stimulatory [1]. A hallmark of human KIR is their variegated expression pattern on subsets of NK cells [2], [3] and T cells [4] and specificity for their ligands, the highly polymorphic HLA class I proteins [5]. Combinations of inherited *KIR* and *HLA class I* genes essentially influence functional maturation of human NK cells [1], [6], susceptibility to infectious [2], [3], [7]–[9] and autoimmune diseases [4], [10], various types of cancer [5], [11], and reproduction [12].

Macaques are used as important nonhuman primate models to study these diseases and, hence, the role of KIR. It was shown recently that rhesus macaque *KIR* genes and haplotypes are at least as diverse as their human counterparts [13]–[16]. With the exception of KIR2DL4, KIR2DL5 and KIR1D, all rhesus macaque KIRs consist of three Ig domains [17]. Further differences between human and macaque KIR are evident in the structure of macaque activating KIR that combine characteristics of KIR3DL and KIR2DL4 molecules [18]. *KIR3D* genes have undergone enormous expansions and diversifications in macaques [13]–[16], [19], [20] and their encoded proteins specifically interact with HLA-A-related Mamu-A MHC class I proteins in a locus and allele-specific manner [21]. Recent studies reported contributions of inhibitory and activating *KIR* genes with viral load in simian immunodeficiency virus (SIV) experimental infection in rhesus macaques [22]–[24].

Despite these efforts to characterize *KIR* genes and their expected importance in rhesus macaque disease models, no data has been published so far on KIR protein expression due to non-availability of specific antibodies. We have recently established and characterized monoclonal antibodies against rhesus macaque KIR proteins [25]. Here, we used these monoclonal antibodies to study the expression of KIR proteins on subsets of NK and T cells in rhesus macaques and other Old World monkeys. We found clonal expression patterns of KIR proteins on NK cell and T cell subsets in rhesus macaques. In contrast to human CD56bright NK cells, the corresponding rhesus macaque NK-cell subset expresses KIR proteins. Analysis of a small number of animals expressing KIR3DL05, a KIR with known MHC class I specificity, did not show any influence of the presence of the ligand on the frequency of KIR3DL05-expressing NK cells.

## Methods

### Ethical statement

Blood sampling procedures were conducted at the German Primate Center in Göttingen. The studies were performed in accordance with the German Animal Welfare Act (Tierschutzgesetz der Bundesrepublik Deutschland 25.05.1998). This includes supervising and advice by the institutional animal welfare officer and approval by the governmental veterinary authorities. The corresponding reference number of the approval for blood sampling is 33.9-425-05-10A102 given by LAVES (Lower Saxony State Office for Consumer Protection and Food Safety). LAVES is the regional governmental veterinary authority that is responsible for the allowance of animal experiments in Lower Saxony. The ongoing of the procedures were controlled and supervised by the local and regional veterinary authorities, the veterinary staff and the animal welfare officer of the German Primate Center. The animals are kept under conditions documented in the European Directive 2010/63/EU (directive on the protection of animals used for experimental and other scientific purposes) and the EU Recommendations 2007/526/EG (guidelines for the accommodation and care of animals used for experimental and other scientific purposes). These conditions are consistent with the regulations of the Guide for Care and Use of Laboratory Animals by the National Research Council (USA). The three Rs are considered using the 3Rs Guidelines for Primate Accommodation, Care and Use by the National Centre for the Replacement, Refinement and Reduction of Animals in Research (UK).

The blood samples were obtained in combination with the annual health monitoring tests of the German Primate Center breeding colonies or in combination with necessary veterinary procedures in stock animals. Blood samples were taken under appropriate narcosis (ketamine, xylazin). None of the animals were euthanized. The procedures were performed in accordance with the described regulations of the local and regional veterinary authorities and under attention of the national and European animal welfare regulations (EU directive 2010/63 EU, German Animal Welfare Act). The institutional animal welfare officer, who has to agree to the procedure, was informed prior to the blood withdrawal.

Housing conditions, enrichment, feeding:

Rhesus macaques

1. housing conditions:
  - indoor and outdoor cage for one breeding group
  - indoor cage with 43 sqm and 7 m height; heated 18–20 C; wood bedding on the ground
  - outdoor cage with 370 sqm and 7 m height; natural ground
  - enrichment with wood (bar, hut), ropes, firehoses, car tires, barrels, chain
2. feeding:
  - three times per day in changing composition, fresh water ad libitum
  - fruits, vegetables, special primate pellets, seeds
  - cooked potatoes, eggs, rice

Hamadryas baboons

1. housing conditions
  - indoor rooms and outdoor cage for one breeding group
  - indoor room: 27 sqm and 2.50 m height, heated 18–20 C, wood bedding on the ground
  - outdoor cage with 85 sqm and 3.5 m height, bark on the ground
  - enrichment with wood (bar, hut), ropes, firehoses, car tires, barrels, chain
2. feeding:
  - three times per day in changing composition, fresh water ad libitum
  - fruits, vegetables, special primate pellets, seeds,
  - cooked potatoes, eggs, rice

Cynomolgus macaques

1. housing conditions:
  - indoor and outdoor cage for one breeding group
  - indoor cage with 40 sqm and 3 m height; heated 20–23 C; wood bedding on the ground
  - outdoor cage with 141 sqm and 4 m height; bark on the ground
  - enrichment with wood (bar, hut), ropes, firehoses, car wheels, barrels, chain
2. feeding:
  - three times per day in changing composition, fresh water ad libitum
  - fruits, vegetables, special primate pellets, seeds
  - cooked potatoes, eggs, rice

African green monkeys

1. housing conditions:
  - experimental primate cage with 1 sqm and 2 m height for one animal
  - the cages are connected together at a group cage
  - enrichment with wood, chain and toys special for primates
2. feeding:
  - three times per day in changing composition, fresh water ad libitum
  - fruits, vegetables, special primate pellets, seeds
  - cooked potatoes, eggs, rice

### Cell samples

Peripheral blood was obtained from 8 rhesus macaques (*Macaca mulatta*), 4 cynomolgus macaques (*Macaca fascicularis*), 9 hamadryas baboons (*Papio hamadryas*) and 4 African green monkeys (*Chlococebus sabaeus*) that are housed at the German Primate Center. Peripheral blood mononuclear cells (PBMC) were isolated from heparinised blood using a ficoll density gradient. The blood was diluted 1∶1 with RPMI medium and 30 ml were transferred to a Leucosep® tube containing 15 ml ficoll and centrifuged for 40 min with 800 x g. The PBMC layer was transferred to a new tube and washed with RPMI medium.

### Antibodies and flow cytometry

The following monoclonal antibodies and labels were used for analysis of PBMCs: Alexa Fluor 700-conjugated CD3 (clone SP34-2, BD), V450-conjugated CD4 (clone L200, BD), V500-conjugated CD8 (clone RPA-T8, BD), APC-conjugated CD11c (clone B-ly6, BD), PERCP-Cy5.5-conjugated CD14 (clone M5E2, BD), APC-Cy7-conjugated CD16 (3G8, BD), PE-Cy7-conjugated CD20 (clone L27, BD), FITC-conjugated CD56 (clone NCAM16.2, BD), PE-conjugated CD159a (NKG2A, clone Z199, Beckman Coulter), FITC-conjugated γδTCR (clone SA6.E9, Invitrogen). Monoclonal antibodies 1C7 (pan-KIR), 2H5 (KIR3DL05 specific), and 2H9 (KIR3DLW03, KIR3DS05) [25] were labelled with DyLight633 and DyLight488 (1C7, 2H5) using the DyLight antibody labelling kit according to the manufacturer’s recommendations (Thermo Scientific).

For each sample 1−2×10^6^ cells were stained and used for flow cytometry. The PBMC samples were incubated with one of the three antibody mixtures in addition to either DyLight633-conjugated 1C7, 2H5 or 2H9: (1) KIR-NK cell I: CD3, CD8, CD14, CD16, CD20, CD159a, (2) KIR-NK cell II: CD3, CD8, CD14, CD16, CD20, CD56, CD159a, (3) CD3, CD4, CD8, CD14, CD16, CD20, CD159a, γδTCR for 30 min at 4°C, fixed with 3.5% formaldehyde in FACS-buffer for 10 min at RT and centrifuged for 5 min (200 x g). The cell pellets were resuspended in 50 µl FACS buffer, measured in a LSR II (BD) and analysed using FlowJo 8.8.7 software. Correlation between frequency of KIR expression and mean fluorescence intensity (MFI) was analysed by linear regression using Prism software version 4.0.

### Identification of *MHC class I* and *KIR* gene transcripts

Total RNA was extracted from PBMCs with the RNeasy Plus Mini KIT (Qiagen). *MHC class I* and *KIR* gene transcripts of rhesus macaques were analysed from respective cDNA samples using Roche/454 Titanium chemistry in a GS Junior sequencer (Roche Applied Science) according to previously published methods [16], [26] and manufacturer’s recommendations. Pools of 8 samples were sequenced on single PicoTiterPlates with respective MID-tagged primers.

## Results

### KIR protein expression on rhesus macaque NK cells

**Figure 1:**
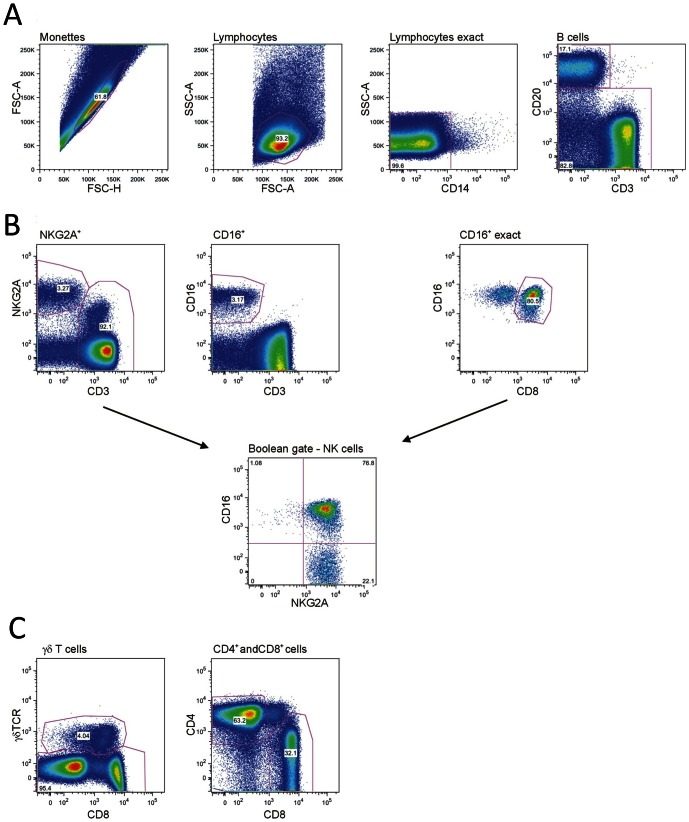
Gating strategy for rhesus macaque PBMCs. (A) After gating on monettes (FSC-A, FSC-H) and lymphocytes (SSC-A, FSC-A), CD14-positive cells and CD20^+^ (B cells) were excluded. (B) NK cells and T cells were identified based on NKG2A/C, CD16 and CD3 expression. CD16-positive mDCs were excluded by gating on CD8-positive CD16-positive cells (CD16^+^ exact gate). A Boolean gate was subsequently produced to display the NK cells. (C) The CD3-positive cells were distinguished based on γδ TCR, CD4, and CD8 expression.

We used the gating strategy outlined in Figure 1 to analyse rhesus macaque NK cells. First, all doublets were excluded using the FSC-H against FSC-A (monettes), granulocytes and most of the monocytes were separated by size using the SSC-A against the FSC-A (lymphocytes). The lymphocyte population was then controlled for remaining monocytes by using CD14 (lymphoctes exact) and was further analysed with CD20 (B cells) and CD3 (T cells) to exclude the CD20^+^ B cells and the remaining CD20^−^ and CD14^−^ population was used to characterise the NK cells and different T cell subsets (Figure 1A). The traditional human NK cell marker CD56 is not suitable to characterise rhesus macaque NK cells derived from PBMC samples as this marker is expressed mainly by monocytes [27] and only about 2% of all rhesus macaque blood NK cells [28]. However, almost all (97–98%) rhesus macaque NK cells are positive with monoclonal antibody Z199 [29] that in rhesus macaques detects both NKG2A and NKG2C [30]. Therefore, we defined rhesus macaque NK cells as CD3^−^ NKG2A/C^+^. All NK cells also express CD8 [28] and many are positive for CD16. Therefore, these markers were used in combination with NKG2A/C. As myeloid dendritic cells (mDC) also express CD16 in rhesus macaques [31], all CD16-positive cells had to be further analysed by gating these cells against CD8 to exclude the CD8-negative mDCs (CD16 exact population). A Boolean gate was then generated with the NKG2A/C-positive population and the CD16 exact population and these cells were defined as NK cells (Figure 1B). T cell subsets were analysed based on markers CD3, CD4, CD8 and γδ TCR (Figure 1C). As a result of these analyses, 3–15% NK cells and 30–70% T cells were identified in blood lymphocytes of the 8 studied rhesus macaques. The frequencies of T cell subsets were: 37–75% CD4^+^αβ T cells, 17–47% CD8^+^αβ T cells and 2–15% γδ T cells (not shown).

**Figure 2:**
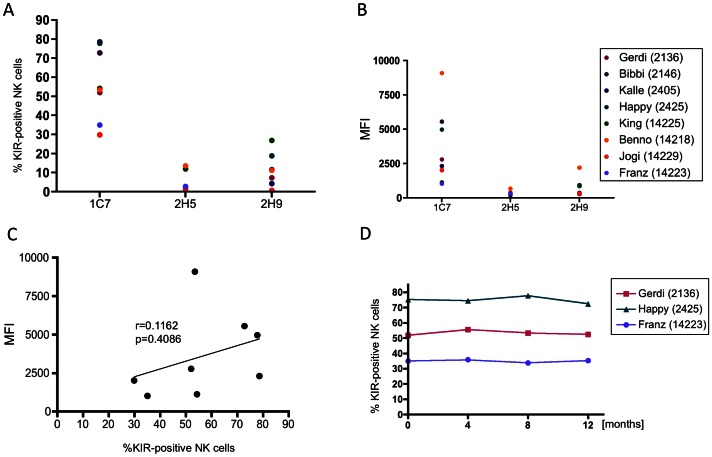
Analysis of KIR3D protein expression on rhesus macaque NK cells. (A) % KIR-positive NK cells and (B) mean fluorescence intensity (MFI) are shown for antibodies 1C7 (pan-KIR3D), 2H5 (KIR3DL05), and 2H9 (KIR3DLW03, KIR3DS05) for the indicated rhesus macaques. (C) Linear regression analysis of % KIR-positive NK cells and mean fluorescence intensity is shown for antibody 1C7. (D) Frequencies of KIR3D-positive NK cells (antibody 1C7) were followed in three animals over a period of 12 months in 4-month-intervalls. Antibody 2H9 detects KIR3DS05 and KIR3DLW03 in animal 2425 and only KIR3DS05 in animals 2136, 2146, 2405, 14225, and 14218, and only KIR3DLW03 in animal 14229 according to *KIR* genotyping (see Table 1).

Expression of KIR3D molecules on NK cells was monitored by pan-KIR antibody 1C7 [25] in the eight rhesus macaques. The percentage of KIR-positive NK cells varied between 29.9 and 78.6% in the analysed rhesus macaques (Figure 2A). Similarly, also the amount of KIR on the NK cell surface differed (Figure 2B), but there was no correlation between the frequency of KIR-expressing NK cells and MFI (r = 0.1162, p = 0.4086; Figure 2C). The frequency of KIR-expressing NK cells was followed in three animals over a period of one year and turned out to be constant (Figure 2D).

**Figure 3:**
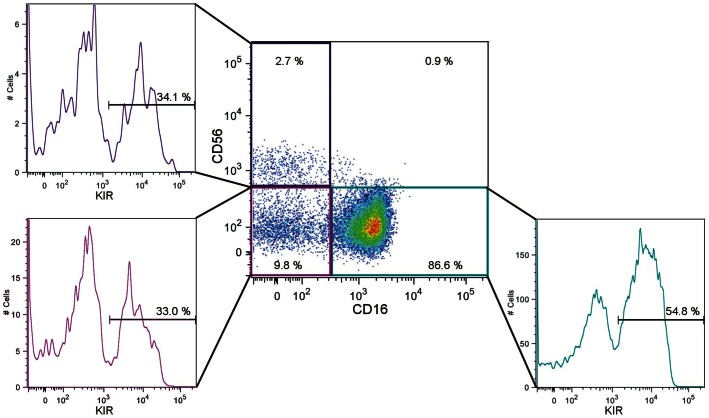
Analysis of KIR3D protein expression on rhesus macaque NK cell subsets. NK cells were analysed for CD56, CD16 and pan-KIR3D antibody 1C7. Results obtained from rhesus macaque individual Gerdi (2136) are shown. All three NK cell subsets express KIR3D proteins: CD56^+^CD16^−^ (34.1%), CD56^−^CD16^−^ (33.0%), CD56^−^CD16^+^ (54.8%).

Three different subsets of rhesus macaque NK cells can be defined with markers CD56 and CD16 in rhesus macaques: CD56^+^CD16^−^ (1.6 – 12%, n = 4, mean 4.9%), CD56^−^CD16^−^(5.6 –16%, n = 4, mean 9.7%), and CD56^−^CD16^+^ (69–92%, n = 4, mean 83%) (Figure 3). These subsets correspond to human CD56^bright^CD16^−^, CD56^dim^CD16^−^, and CD56^dim^CD16^+^ [32]. The three subsets were also analysed with DyLight633-labelled pan-KIR3D antibody 1C7 and KIR expression was found on all three NK cell subsets: 18% (15–34%) of the CD56^+^CD16^−^ subset, 40% (30–57%) of the CD56^−^CD16^−^subset, and 73% (55–84%) of the CD56^−^CD16^+^ subset.

We also stained rhesus macaque NK cells with DyLight633-labelled 2H5, an antibody that exclusively binds rhesus macaque KIR3DL05 [25]. Individuals differ in the frequency of KIR3DL05 expression, ranging between 1.5 and 15.6% KIR3DL05-positive NK cells (Figure 2A; Table 1). The MFI of expressed KIR3DL05 varied between 189 and 662 for the tested animals (Figure 2B; Table 1). No correlation between frequency of 2H5-positive NK cells and MFI was found (r = 0.2479, p = 0.3933). DyLight633-labelled antibody 2H9, which detects rhesus macaque KIR3DLW03 and KIR3DS05 [25], was tested in seven rhesus macaques and reacts with 0.8–26.9% of NK cells (Figure 2A). Interestingly, all animals are genotyped as positive for KIR3DS05 except animal 14229, and only animals 2425 and 14229 have KIR3DLW03. Thus, the percentages seen for animals 2136, 2146, 2405, 14225 and 14218 (Figure 2B) are solely attributable to KIR3DS05 expression, the percentage shown for animal 14229 corresponds to KIR3DLW03, and the value shown for 2405 corresponds to both KIR3DLW03 and KIR3DS05. There was no correlation seen between 2H9-positive NK cells and MFI (r = 0.1215, p = 0.4435).

**Table 1:** Identified *MHC class I* and *KIR* gene transcripts.

Animal number	Mamu-A^a^	Mamu-B	KIR^a^	% KIR3DL05-positive NK cells/MFI^b^	number of different 3DL05 sequences^c^
2405	A1*004; A1*008; A3*13 ; A4*14	B*012; B*021; B*022; B*028; B*030; B*045; B*046; B*060; B*072; B*074; B*097; B*098	*3DL01; 3DL02; * ***3DL05*** *; 3DL07; 3DL10; 3DL11; 3DS02; 3DS05*	2.8/189	1
14218	A1*004; A4*14	B*001; B*007; B*030; B*072	*1D; 3DL01; * ***3DL05*** *; 3DL07; 3DL10; 3DS02; 3DS04; 3DS05; 3DS06*	15.6/662	2
14223	A1*002 ; A1*004; A4*14	B*001; B*007; B*030, B*072	*1D; 3DL01; 3DL04; * ***3DL05*** *; 3DL07; 3DL10; 3DS02; 3DS05*	2.8/310	2
14225	A1*006; A1*028; A4*14	B*012; B*024; B*026; B*030; B*038; B*046; B*057; B*072; B*074; B*082; B*098	*1D; 3DL01; 3DL04; * ***3DL05*** *; 3DL07; 3DL10; 3DS02; 3DS05; 3DSW09*	12.1/229	2
14229	A1*004; A1*041; A4*14	B*001; B*007; B*012; B*030; B*036; B*037; B*045; B*050; B*63; B*072; B*078	*1D; 3DL01 3DL02 3DLW03; * ***3DL05*** *; 3DL07; 3DL08; 3DL11; 3DS02; 3DSW08*	1.5/377	1

a MHC class I proteins that are known to interact with KIR3DL05 are shown in bold type.

b % KIR3DL05-positive NK cells were determined with specific antibody 2H5; mean flourescence intensity (MFI) is indicated.

c in those cases where only a single *3DL05* sequence was found, this might be either due to *3DL05* being present on only one chromosome (1 gene copy) or 3DL05 being present on both chromosomes (2 gene copies, but homozygosity).

We used DyLight488-conjugated 2H5 and DyLight633-conjugated 2H9 antibodies to determine the level of co-expression of KIR3DL05 and KIR3DS05, respectively, in NK cells of animal 2405. 2.0% and 2.5% of all NK cells express KIR3DL05 and KIR3DS05, respectively, and both proteins were detected on 0.85% of NK cells (data not shown). These data confirm the variegated expression patterns of KIR on rhesus macaque NK cells.

In summary, rhesus macaque individuals differ in the frequency of KIR3D-expressing NK cells as well as in the strength of KIR3D protein expression and show a clonal pattern of KIR expression on NK cells, a result that is similar to human KIR-expressing NK cells. Contrasting human CD56^bright^CD16^−^ NK cells are corresponding rhesus macaque CD56^+^CD16^−^NK cells that express KIR with considerable frequency.

### KIR protein expression on rhesus macaque T cell subsets

**Figure 4:**
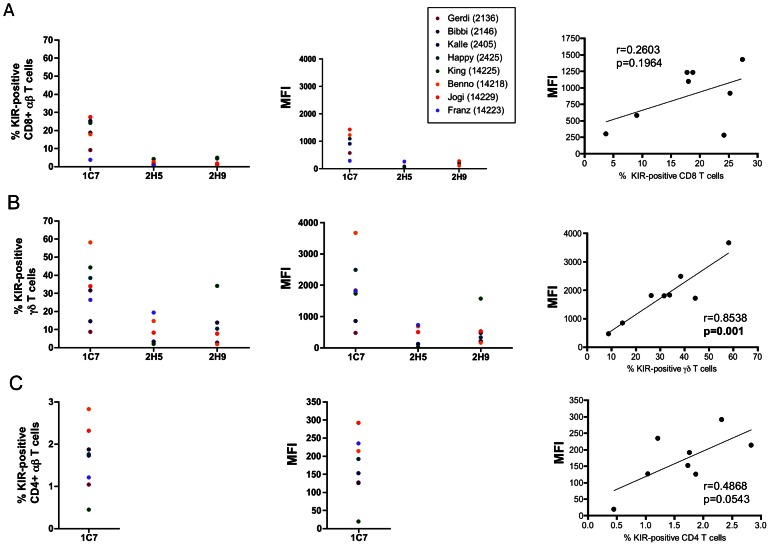
Analysis of KIR3D protein expression on rhesus macaque T cell subsets. (A) CD8 αβ T cells were analysed for frequency of KIR expression (left panel), mean fluorescence intensity (antibody 1C7) (middle panel), linear regression analysis of KIR frequency and MFI (right panel). (B) γδ T cells were analysed for frequency of KIR expression (left panel), mean fluorescence intensity (antibody 1C7) (middle panel), linear regression analysis of KIR frequency and MFI (right panel). (C) CD4 αβ T cells were analysed for frequency of KIR expression (left panel), mean fluorescence intensity (antibody 1C7) (middle panel), linear regression analysis of KIR frequency and MFI (right panel). Antibody 2H5 detects KIR3DL05, whereas antibody 2H9 detects KIR3DS05 and KIR3DLW03 in animal 2425 and only KIR3DS05 in animals 2136, 2146, 2405, 14225, and 14218, and only KIR3DLW03 in animal 14229 according to *KIR* genotyping.

Next we analysed rhesus macaque T cell subsets for KIR expression with DyLight633-labelled pan-KIR3D antibody 1C7. Rhesus macaque CD8^+^αβ T cells express KIR3D proteins with different frequencies (3.7–25.3%) in individuals (Figure 4A). The amount of expressed KIR3D varies, but no correlation between the numbers of KIR3D-positive CD8^+^αβ T cells and the density of expressed KIR3D was noticed (Figure 4A). Antibody 1C7 stained between 8.7–58.1% of all γδ T cells (Figure 4B) and 0.5–2.8% of CD4^+^ αβ T cells (Figure 4C). In contrast to αβ T cells (CD4: r = 0.4868, p = 0.0543; CD8: r = 0.2603, p = 0.1964), there is a statistically significant positive correlation (r = 0.8538, p = 0.001) between the frequency of KIR3D-positive cells and the density of KIR3D on the surface of γδ T cells (Figure 4B).

KIR3DL05 is expressed on 0.8–4.1% of CD8^+^αβ T cells and by 2.0–19.4% of γδ T cells and KIR3DLW03/KIR3DS05 was detected on 0.6–4.9% of CD8^+^ αβ T cells and on 1.9–34.1% of γδ T cells using antibodies 2H5 and 2H9, respectively (Figure 4A). Comparison of the detected percentages for single KIRs with the total KIR expression of both CD8^+^αβ and γδ T cells showed a clonal expression pattern similar to NK cells. Furthermore, the amount of expressed KIRs differs between animals. No correlation between percentage KIR-positive cells and MFI was observed for KIR3DLW03/KIR3DS05 (r = 0.2985, p = 0.2045) or KIR3DL05 (r = –0.2609, p = 0.3791) in CD8^+^αβ T cells, but was noticed for both KIR3DLW03/KIR3DS05 (r = 0.9427, p = 0.0003) and KIR3DL05 (r = 0.9002, p = 0.0138) for γδ T cells.

In summary, KIR3D proteins are expressed in a clonal manner on subsets of rhesus macaque T cells with frequencies similar to human T cells. In contrast to NK cells and αβ T cells, the strength of KIR expression (MFI) and the frequency of KIR-positive cells are positively correlated in γδ T cells.

### KIR protein expression on NK cells and T cell subsets of other Old World monkey species

PBMC samples of other nonhuman primates were analysed for cross-reactivity with the pan-KIR3D antibody 1C7. The same gating strategy as for rhesus macaque PBMCs was applied for PBMCs derived from baboons, cynomolgus macaques and African green monkeys, except for the “exact CD16^+^” population. CD11c-APC was used as an additional marker to exclude mDCs because the CD16/CD8 gating did not work for all tested monkey species (data not shown). Therefore, 1C7 antibody had to be used conjugated with DyLight488, but this excluded the possibility to study γδ TCR in these species using the FITC-conjugated anti-γδ TCR antibody.

**Figure 5:**
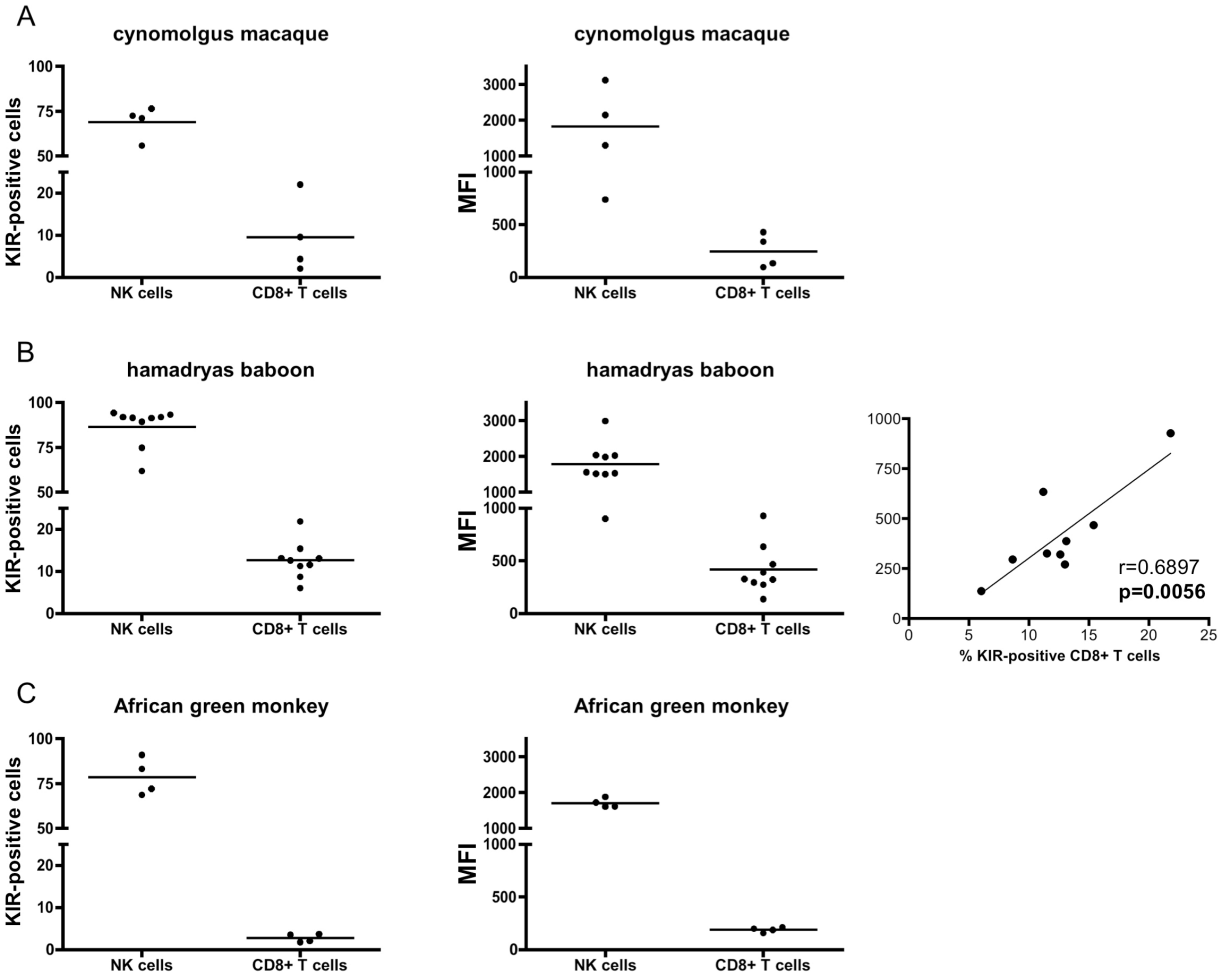
KIR3D protein expression on NK cells and CD8^+^ T cells in other Old World monkeys. Antibody 1C7 was used to determine the frequencies of KIR3D-positive cells (left panels), MFI (middle panels), and linear regression analysis (right panel) in (A) cynomolgus macaques (n = 4), (B) hamadryas baboons (n = 4), and (C) African green monkeys (n = 4).

KIR3D was detected in all tested animals, indicating that pan-KIR3D antibody 1C7 is cross-reactive with KIR of other Old World monkey species. 55.7–76.4% of all cynomolgus macaque NK cells express KIR3D and 2.0–22.0% of CD8^+^αβ T cells (Figure 5A). The frequency and density of expressed KIRs neither correlate for NK cells (r = 0.5054, p = 0.2891) nor for CD8^+^αβ T cells (r = 0.8710, p = 0.0667).

In hamadryas baboons 61.7–94.4% of all NK cells express KIR3D and also the amount of expressed KIR (MFI) varies between different animals. No significant correlation between KIR-positive NK cells and the level of expression could be found (r = 0.2774, p = 0.1452). CD8^+^αβ T cells of hamadryas baboons also express KIR3D (6.0–21.8%) and frequency and amount of KIR correlates significantly (r = 0.6897, p = 0.0056; Figure 5B).

Between 68.7–90.9% of African green monkey NK cells are positive with the pan-KIR antibody 1C7 (Figure 5C). Surprisingly, only 1.8–3.7% of African green monkey CD8^+^αβ T cells express KIR3D. The frequency does not correlate with the expressed density of KIR on NK cells (r = 0.0172, p = 0.8689) or CD8+ T cells (r = 0.64, p = 0.2).

In summary, KIR3D expression can be detected with antibody 1C7 in other Old World monkey species and shows a pattern similar to rhesus macaques. African green monkeys show a remarkably low frequency of CD8^+^αβ T cells that express KIR3D proteins. The frequency of KIR-expressing CD8^+^αβ T cells in hamadryas baboons is correlated with MFI of KIR3D protein expression.

### Influence of MHC class I ligands on frequency of specific KIR-expressing NK cells in rhesus macaques

We determined the transcribed *MHC class I* and *KIR* genes in the 8 studied rhesus macaques by next-generation sequencing according to published protocols [16], [26] to identify animals carrying both KIR and cognate MHC class I ligands. KIR3DL05 is currently the only inhibitory KIR for which both a specific antibody and knowledge of MHC class I ligand specificity is available. Antibody 2H5 specifically detects KIR3DL05 [25], which interacts with Mamu-A1*001 and Mamu-A1*002 as well as Mamu-A3*13 MHC class I proteins [21]. Table 1 lists the five animals that transcribe the *KIR3DL05* gene. Although not formally shown, we do not expect binding of KIR3DL05 to MHC class I allotypes other than Mamu-A1*001, Mamu-A1*002, and Mamu-A3*13 based on the recognition motif of KIR3DL05 [21]. Similar to KIR and HLA in human, we expected to find higher frequencies of expressed KIR in those rhesus macaques where the cognate ligand is also present. Animals 14223 and 2405 express the specific ligands Mamu-A1*002 and Mamu-A3*13, respectively (Table 1), yet we found KIR3DL05 expressed on only 2.8% NK cells in both animals (Table 1). Notably, animal 14218 expresses almost the same *MHC class I* alleles as 14223 except for Mamu-A1*002, but shows 15.6% KIR3DL05-positive NK cells (Table 1). Thus, the presence of cognate ligands obviously has only a minor if any influence on the frequency of KIR3DL05 on blood NK cells in rhesus macaques.

## Discussion

KIR protein expression has been shown to essentially regulate the activity of NK cells. While these data have been collected in human, no such studies could be made in important nonhuman primate disease model organisms such as macaques due to absence of suitable anti-macaque KIR antibodies. Moreover, lack of suitable anti-KIR antibodies prevented basic studies such as KIR-expressing lymphocyte populations in nonhuman primates. We have recently established a panel of mouse monoclonal antibodies raised against rhesus macaque KIR3D proteins [25], which we have now used to address this question in Old World monkey lymphocyte populations.

Rhesus macaques express *KIR2DL4* and *KIR3D* genes, and in some cases also *KIR1D* [16], [33], a lineage III *KIR* gene of unknown biological relevance. As *KIR3D* genes are enormously expanded in macaques [13], [15]–[17], [19], the vast majority of transcribed *KIR* genes are of the *KIR3D* type. We took advantage of our antibody 1C7 that broadly reacts with all tested activating and inhibitory KIR3D proteins (pan-KIR3D antibody) [25] to study the cellular distribution of KIR on rhesus macaque PBMCs. We noticed KIR3D expression on 30–79% of NK cells, 0.5–3% of CD4^+^αβ T cells, 4–25% of CD8^+^αβ T cells, and 9–58% of γδ T cells. Human PBMCs exhibit a similar pattern of KIR protein expression [34]–[37]. This finding is of importance when rhesus macaques are used as animal models of human diseases where NK cells (in particular KIR proteins expressed on NK cells), play important roles such as in SIV infection [22], [23]. Notably, we demonstrated for the first time that KIR proteins show clonal expression patterns on both NK cells and T cell subpopulations in rhesus macaques and other Old World monkeys, further emphasising the functional similarities of KIR in humans and biomedically highly relevant nonhuman primates. Contrasting both macaque species and baboons are limited numbers of KIR-positive CD8^+^αβ T cells in African green monkeys. Despite the limited knowledge of the biological role of KIR expressed on T cells as compared to NK cells, this finding is interesting in the context of simian immunodeficiency virus infection as African green monkeys are natural hosts of SIV and resistant to SIV-induced pathogenesis [38].

However, there are also differences between rhesus macaques and human KIR-expressing NK cells. All rhesus macaque NK cells express the CD94/NKG2A and/or CD94/NKG2C heterodimer. Thus, in contrast to human NK cells that loose NKG2A upon acquisition of KIR expression during NK cell differentiation [2], [3], [39], there is obvious co-expression of NKG2A/NKG2C and KIR on roughly 75% (55–84%) of mature (CD3^−^CD16^+^CD56^−^) blood NK cells in rhesus macaques. The reason for these two extremes might lie in differences in NK cell education (alias licensing or functional maturation). While education of human NK cells is more or less focussed on the KIR2DL1 and KIR2DL2/3 receptors and the HLA-C1/C2 ligands, we speculate that the rhesus macaque KIR/ligand system is probably too variable to allow such focussing in the light of the enormous copy-number variation of both *KIR3D* [13]-[15] and *MHC class I Mamu-A* and *Mamu-B* genes [40]–[42]. In addition, protein interactions between macaque inhibitory and activating KIRs and their specific *Mamu-A1* and *Mamu-A3*-encoded ligands are characterised by lower avidity and broader reactivity [21], [43] compared to the human KIR/ligand systems. These factors, high gene-content variability of both KIR and ligand system, lower avidity and broader specificity, might contribute to less impact of (inhibitory) KIR and to a greater relevance of the conserved CD94/NKG2A receptor in the education of NK cells to detect ‘missing self’ in rhesus macaques. The hominid (great apes and human) KIR system underwent an expansion of lineage III *KIR* genes encoding KIR2D receptors with a D1–D2 domain organisation functionally focussed on the emerged MHC-C ligand [44], [45]. This focussed co-evolution of KIR and ligand is particularly evident in human [46], but must not necessarily be evident in other primates.

Rhesus macaques differ in the frequency of γδ T cells that express KIR3D proteins, ranging from 9 to 58% of KIR-positive γδ T cells in the animals studied here. In animal 14225 (King) 44.3% and 34.1% of the γδ T cells react with antibodies 1C7 (pan-KIR3D) and 2H9 (3DLW03, 3DS05), respectively (Figure 4B). Because 14225 expresses KIR3DS05 but not KIR3DLW03 (Table 1), one third of all blood γδ T cells express the stimulatory KIR3DS05 in this animal. Currently, we do not have further anti-KIR antibodies with single specificity available to determine the exact number of blood γδ T cells that only express KIR3DS05. Given that roughly 75% of all 1C7-positive γδ T cells express KIR3DS05, we expect a significant number of γδ T cells only expressing KIR3DS05. TCR-independent stimulation is a known phenomenon of γδ T cells [47] and we expect KIR3DS05 to contribute to this activation in view of its expected prevalent expression in this T cell subset. Surprisingly, we found a strong correlation between the frequency of KIR-expressing cells and the expression level of KIR proteins in γδ T cells in all studied rhesus macaques, which was neither evident in CD4^+^ or CD8^+^ αβ T cells nor in NK cells (Figures 2,4). Thus, factors (e.g. cell education or differentiation) influencing KIR protein expression of γδ T cells are obviously different from αβ T cells and NK cells. Otherwise, this correlation may simply mirror the known perturbations of γδ T cell subsets in rhesus macaques during an immune response [48]. Only in hamadryas baboons we found such a correlation in KIR-expressing CD8^+^αβ T cells, indicating that species can differ in this respect.

Marker proteins CD56 and CD16 can be used to describe three subsets of rhesus macaque blood NK cells: CD56^+^CD16^−^, CD56^−^CD16^−^, and CD56^−^CD16^+^ (Figure 3). Expression profiles of adhesion molecules, cytokines, chemokines and killing activity indicated functional similarity of these rhesus macaque NK cell subsets with human CD56^bright^CD16^−^, CD56^dim^CD16^−^, and CD56^dim^CD16^+^ subsets, respectively [28]. However, as shown here a major difference is that rhesus macaque NK cells do not gradually loose NKG2A (or NKG2C) and acquire KIR expression. Besides stable NKG2A/NKG2C expression, our data demonstrate KIR expression in all three subsets of rhesus macaque NK cells, albeit at somewhat different frequencies. Thus, there are some obvious general similarities in the differentiation of rhesus macaque and human NK cells such as the clonal expression pattern of KIR, whereas the dissimilarities in KIR protein expression during terminal differentiation might be seen as further indication of KIR playing a less important role in rhesus macaque NK cell education (or licensing) as compared to human. Furthermore, the presence of cognate MHC class I ligands obviously has no influence on the frequency of KIR3DL05 protein expression, at least in the rhesus macaques analysed here. Other possible reasons for this lack of influence could lie in the small number of studied animals or in specialised functions of KIR3DL05 that might be independent of MHC class I recognition such as binding of TLR9 ligands, which was reported for human KIR3DL2 [49]. Detailed knowledge on KIR-ligand interaction in macaques is rather limited and is available only for KIR3DLW03, KIR3DL05, KIR3DL11, and KIR3DS05 in rhesus macaques [21], [50] and a single inhibitory KIR3D in pigtailed macaques [43]. Nevertheless, the data presented here should now facilitate detailed studies on KIR on the protein level in experimental animal models that are much closer to human diseases compared to mouse models where the elaborated KIR and ligand system does not exist.
